# Fried sweetpotato user preferences identified in Nigeria and Ghana and implications for trait evaluation

**DOI:** 10.1111/ijfs.14764

**Published:** 2020-09-23

**Authors:** Reuben Ssali, Edward Carey, Simon Imoro, Jan W. Low, Eric Kuuna Dery, Abena Boakye, Ibok Oduro, Rachel M. Omodamiro, Hauwa Ladi Yusuf, Eunice Etwire, Abigail O. Iyilade, Souleimane Adekambi, Abdullahi Ali, Muhammad Haliru, Prince Maxwell Etwire

**Affiliations:** ^1^ International Potato Center (CIP) c/o CSIR‐CRI, P.O. Box 38785, Fumesua Kumasi Ghana; ^2^ International Potato Center (CIP) P.O. Box 25171 Nairobi 00603 Kenya; ^3^ Kwame Nkrumah University of Science and Technology UPO PMB Kumasi Ghana; ^4^ National Root Crops Research Institute P.M.B 7006 Umuahia Umudike Abia State 440001 Nigeria; ^5^ Bayero University P.M.B 3011 Kano Nigeria; ^6^ Independent consultant c/o CSIR‐Savanna Agricutural Institute, P.O. Box TL 52 Tamale Ghana; ^7^ Agricultural and Rural Management Training Institute P. O. Box 4503 Ilorin Kwara State Nigeria; ^8^ University of Parakou 01 BP 123 Parakou Benin; ^9^ CSIR‐Savanna Agricultural Research Institute P.O. Box TL 52 Nyankpala Ghana

**Keywords:** Fried sweetpotato, breeding, profile, characteristics, chunk fries, West Africa

## Abstract

Fried sweetpotato quality is important for variety adoption in West Africa. To inform breeding efforts, the study developed a product profile for sweetpotato chunk fries using mixed qualitative and quantitative methods. Root characteristics, processing attributes, in‐mouth attributes and appearance of fried product were critical to final product quality. Raw roots should be hard, have smooth skin and no off‐odours. Peeled roots should be hard to slice and not sticky. Stickiness and moist surface indicate high moisture content, associated with excessive oil absorption during frying. Hard to slice roots connote high dry matter. Fried product should be crisp, slightly sugary and mealy, have a uniform colour with brown tint and not be soggy. Crispness, mealiness and short frying time with limited oil absorption may be functions of starch. Understanding starch characteristics and other attributes that contribute to quality fried sweetpotato is critical for breeding sweetpotato genotypes with superior quality for frying.

## Graphical abstract

Quality and psychosocial factors influencing the purchase of orangefleshed sweetpotato bread, process for determining drivers for orange‐fleshed sweetpotato bread.

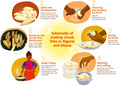

## Introduction

Sweetpotato (*Ipomoea batatas* (L) Lam) is of increasing importance for food and nutrition security in sub‐Saharan Africa (SSA; Low *et al*., 2020). It is a versatile climate‐resilient crop because it is easily propagated and can grow with few external inputs on degraded soils under a range of rainfall patterns (Mukherjee *et al*., 2015). This is an advantage for poor households dependent on diverse livelihood strategies. Sweetpotato varieties grown in SSA include diverse landraces (selected by farmers) and several varieties that are superior with respect to production and nutritional value developed by breeding programmes which have emphasised pro‐vitamin A‐rich orange‐fleshed types during the past two decades (Grüneberg *et al*., 2015; Mwanga *et al*., 2017). Evaluation of sweetpotato germplasm for varietal release typically focuses on agronomic traits that determine yield under biotic and abiotic stress. However, consumer preferences can also determine the success of a newly released variety (Jenkins *et al*., 2018). The high variability in quality attributes and textural properties of fresh and stored sweetpotato roots, coupled with an increasing range of value‐added food products from the crop (Vithu *et al*., 2019), requires that greater attention be paid to understanding the quality characteristics desired by different users along the value chain.

West Africa experiences an extreme climatic gradient, which transitions from the hyper‐arid Sahara Desert to lowland tropical forests (at elevations < 800 m.a.s.l.) with unstable rainy seasons characterised by short to prolonged dry seasons (2–8 months) (Fick & Hijmans, 2017). Sweetpotato is grown in almost all West African countries (FAOSTAT 2018), given its ability to adapt to different environments. It is generally consumed boiled, fried or roasted (Odora *et al*., 2000; Faniyan, 2012), with limited amounts processed into value‐added products like dried chips, flour and puree (Akoroda, 2009; Owade *et al*., 2018). In West Africa, sweetpotato is mostly used as a snack food (Sugri *et al*., 2012), commonly sold in urban centres and rural markets as a fried product in the form of large slices or ‘chunk fries’. These chunk fries are mostly produced by small‐scale, female street vendors. An estimated 30–80% of total sweetpotato production (mostly white or yellow‐fleshed) is fried in parts of Nigeria, Ghana, Burkina Faso and Côte d’Ivoire (Peters, 2015). However, a recent literature review (Carey *et al*., 2021) revealed that fried sweetpotato product research has focused on chips and crisps typically sold in formal markets. The primary objective of this study is to develop a heretofore non‐existent profile of production, processing and consumer‐demanded traits of the widespread ‘chunk fry’ sweetpotato product widely sold in informal markets to ensure that breeding efforts result in suitable varieties for West Africa.

## Materials and methods

### Study area

The study was conducted in September 2019 in Kano and Kwara States in Nigeria, and Bawku Municipality and its environs in the Upper East Region of Ghana, all well‐known major sweetpotato producing areas. To ensure that stakeholders involved would be highly likely to know desired characteristics of the crop, four communities in each of these states/regions were purposively selected. The three dominant local languages found in these study areas are Kusal in Bawku, Yoruba in Kwara State and Hausa in Kano State. Each research team had at least one member fluent in the dominant local language, enabling interviews to be conducted in the local language driven by interviewee preference.

### Study design

A gendered food mapping study was conducted in the study communities in Kano and Kwara States in Nigeria and Bawku. This study relied on a mixed methods research design developed by Forsythe et al. (2021). The study deployed a multistage sampling technique to collect data at various level of sweetpotato production and utilisation. The study followed Forsythe et al. (2021) recommendations on design of multiple survey instruments, quality assurance, and the number of sites and stakeholders required for each process in Ghana and Nigeria. For each State/Region, two local government areas were selected for the study. In each local government area, two communities were selected from which one key informant was selected per community and at least six male and six female participants for focus group discussions. In each community, ten members were randomly selected for the individual interviews. The FGDs also identified urban areas for market information interviews. The study conducted 11 key informant interviews (all male^1^); 22 FGDs involving 200 participants (118 male (M), 82 female (F); 95 individual interviews (64M, 31F); and 24 market‐based interviews involving 60 respondents (33M, 27F). From these data, the first iteration of a fried sweetpotato product profile was developed detailing preferred and non‐preferred characteristics and varieties for fried sweetpotato.

**Figure 1:**
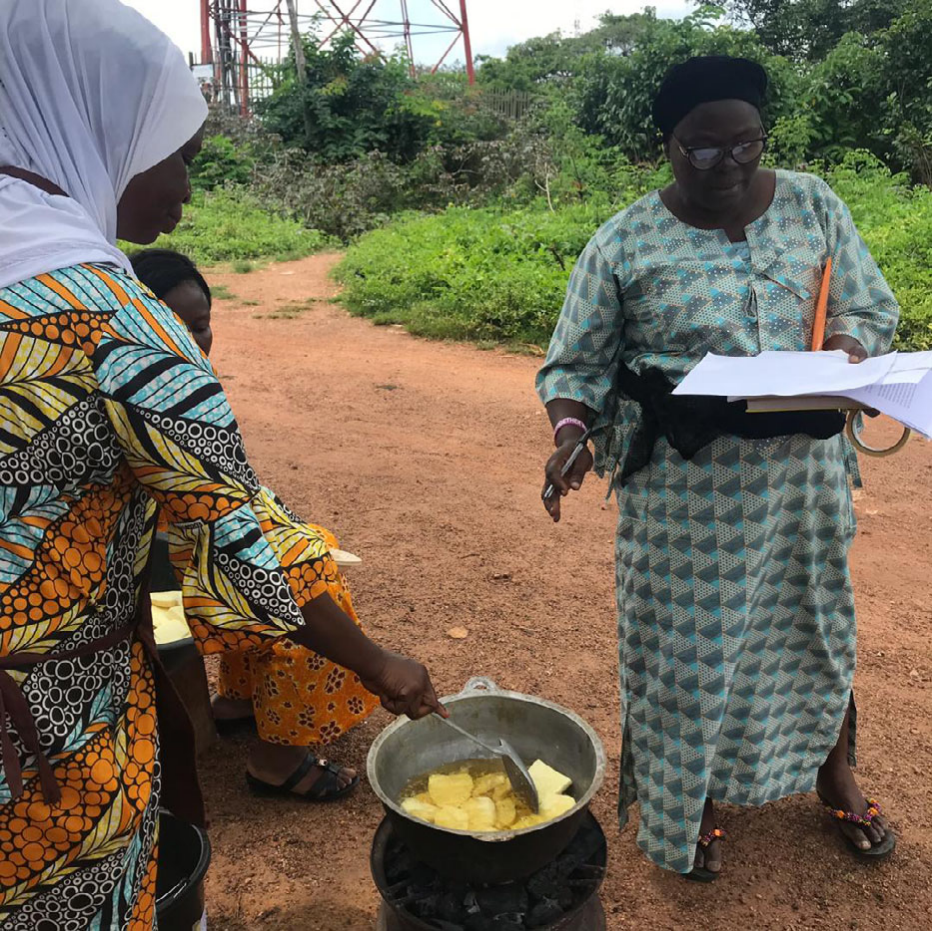
Dr. Rachel Odomamiro interviewing expert chunk fry processor in Agbamu, Kwara State in Nigeria.

Diagnostics on the entire process of producing ‘chunk fries’ were conducted with 20 female processors^2^ (six in Kano, six in Kwara, and eight in Bawku), with 16 different sweetpotato varieties, to evaluate the characteristics of roots needed at each stage (Figure 1). The 16 varieties were selected based on opinions from FGD participants, who specified which were the most preferred varieties (Obare, Alausa, Dan Izala, Dan Bakalori and Dan China), moderately preferred varieties (Purupuru, Dan Barmawa, Dan Madagali, Tomude and Pakurumon) and least preferred varieties (Amuskwera, Dan Silver, Aregbe and Elege) of any flesh type, plus the best orange‐fleshed varieties (Kuffour and Mother's Delight). In addition, the processors selected were considered by FGD respondents to be the best or ‘champion fryers’ in their communities. The varieties found in Ghana differed from those in Nigeria. In Nigeria different varieties were recommended by FGDs in Kwara and Kano sites, except for the orange‐fleshed variety, Mother’s Delight, present in both.

### Data analysis

Qualitative data were coded and analysed using content analysis recommended by Forsythe et al. (2021). Priority rankings for preferred varieties and characteristics were obtained after applying weights to the data. The first, second, third and fourth positions as stated by interviewees were weighted by a factor of 4, 3, 2 and 1, respectively. Subsequently, the rankings of characteristics or varieties were examined by region and gender.

## Results and discussion

### Fried sweetpotato products in the value chain

**Figure 2:**
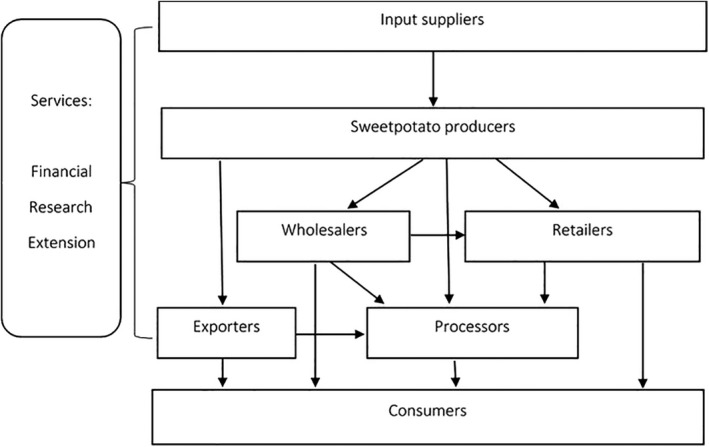
Sweetpotato value chain in Kano and Kwara States in Nigeria and Bawku, the Upper East Region of Ghana.

The market information surveys revealed that the sweetpotato value chain in the study areas included sweetpotato multipliers who provided vines (used as planting material), and agro‐enterprises that provided inorganic fertilisers and other inputs required for sweetpotato production (Figure 2). However, a large number of sweetpotato producers (90%) did not purchase inputs, obtaining their planting material from their own farms or from neighbours, and using manure from their own livestock in lieu of purchased fertiliser. Apart from producers, other agro‐input dealers were located mainly in peri‐urban and urban areas. Production of sweetpotato was mostly done in rural communities. Individual interviews showed that both men and women sold varying proportions of their harvested roots to assemblers, wholesale traders, processors or retailers and exporters. For instance, women in Kwara sold 41% of their produce whilst men sold 74% of the produce suggesting that sweetpotato is a more important commercial crop for men than women, but women also engage in marketing roots. In contrast, similar proportions were sold by both men and women in Kano (98%) and Bawku (80%), making sweetpotato a major commercial crop in these areas. Assemblers and wholesale traders circulated around sweetpotato growing areas, bulking roots for transport. Whereas wholesale and export trading were popular amongst men (93%), processors were exclusively women, and 45% of the root retailers were women. In the major Bawku market, no male processor or retailer was found, reflecting dominant gender roles. Unlike many parts of SSA, a direct relationship between producers and consumers was not common in the study regions, as sweetpotato consumers either produce their own roots or buy them from wholesalers or retailers. In addition to consumers, wholesalers sold their roots to processors and retailers. Financial, research and extension services cut across the entire value chain as these services were needed by all actors. However, access to extension services varied across communities, with the highest access reported by respondents in Kano (73%), compared to only 10% and 3.5% in Bawku and Kwara, respectively. The percentage of men and women reporting access to extension services was similar in all communities.

Farmers did not process before selling their roots. Leaves were rarely marketed. Sweetpotato was regarded highly by men in English‐speaking Bawku, because it is largely transported for sale to French‐speaking Burkina Faso. Given the prohibitive social and economic constraints associated with cross‐border trade, women in Bawku tended to cultivate other crops like maize and soybean for income.

**Figure 3:**
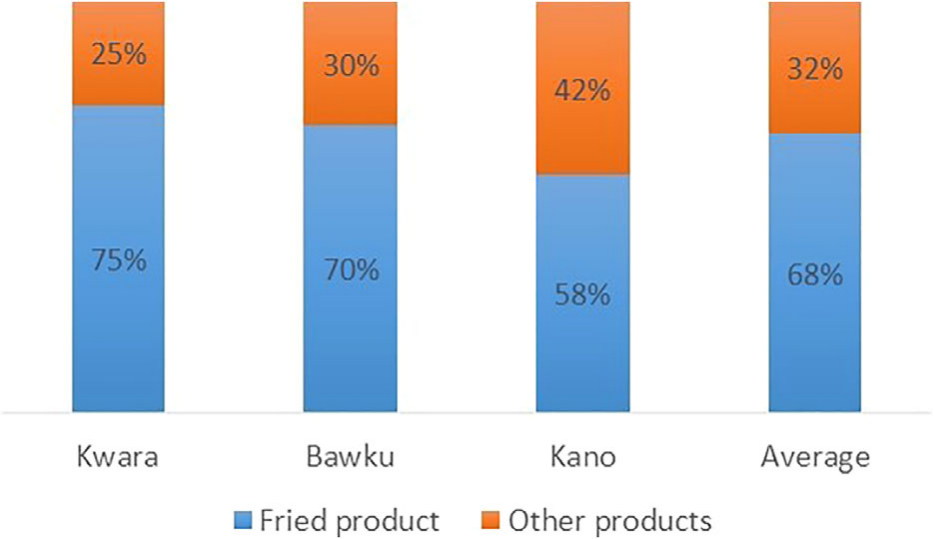
Proportion of sweetpotato consumed as a fried product in Kano and Kwara States in Nigeria and Bawku, the Upper East Region of Ghana.

Generally, all harvested produce was sold as fresh roots in the communities. At the peri‐urban level, traders resold nearly all the roots fresh, with the proportion of roots processed prior to sale not exceeding 10%. Processing was mainly done at the urban centres, where up to 75% of roots purchased by processors were made into fries and other products before sale (Figure 3). This highlights the need for farmers to grow varieties suitable for frying and with low perishability during transportation to ensure markets for their produce.

### Main sweetpotato varieties grown by farmers

Households in each community grew from four to eleven sweetpotato varieties. In all the communities studied, men and women expressed different reasons for growing specific varieties (Table 1). The most preferred variety by women in Kano State was the local landrace Dan Izala (white‐fleshed) because of its high yield, short maturity period (<4 months) and better market price. Men grew Dan Izala for its early maturity and drought tolerance (Table 1). White‐fleshed local landrace Dan Bakalori was the most preferred variety by men in Kano because of its short maturity period, but women reported growing it for its good taste and flesh softness. Another variety regarded highly by both men and women in Kano was the white‐fleshed local landrace Dan China. Men reported growing Dan China for its high yield, whilst women cited its good taste and high market value.

**Table 1:** Reasons mentioned for growing specific sweetpotato varieties in Kano and Kwara States in Nigeria and Bawku in Upper East Region of Ghana by gender

Reasons why varieties are preferred	% of women citing (n = 31)	% of men citing (n = 64)	Number of varieties (Names: Ka (if in Kano), Kw (if in Kwara), Ba (if in Bwaku)
Easily marketable	50.9	39.9	4 (Pakurumon‐Kw, Alausa‐Kw, Aragbe‐Kw, Mother’s Delight‐Kw)
Good taste – sugariness/less sweet	50.8	26.5	7 (Dan Izala‐Ka, Dan China‐Ka, Dan Bakalori‐Ka, Pakurumon‐Kw, Aragbe‐Kw, Obare‐Ba, Apomuden‐Ba)
Bigger root size	50	27.1	3 (Dan Izala‐Ka, Dakata‐Ka, Dan Barmawa‐Ka)
High yielding	48.7	65.7	10 (Dan Izala‐Ka, Dan China‐Ka, Dan Bakalori‐Ka, Dan Barmawa‐Ka, Pakurumon‐Kw, Aregbe‐Kw, Mother’s Delight‐Kw, Obare‐Ba, Purupuru‐Ba, Amuskwera‐Ba)
Long vines for feeding animals	41.7	0	2 (Dan Izala‐Ka, Dan Barmawa‐Ka)
Short cooking time	37.5	0	1 (Dakata‐Ka)
Better market price	36.7	0	2 (Dan Izala‐Ka, Dan China‐Ka)
Mealiness	35.0	0.9	2 (Dan Izala‐Ka, Dan Barmawa‐Ka)
Less water content	33.0	6.3	1 (Dan Bakalori‐Ka)
Early maturity	30.8	69.9	9 (Dan Izala‐Ka, Dan China‐Ka, Dan Bakalori‐Ka, Dan Barmawa‐Ka, Pakurumon‐Kw, Alausa‐Kw, Mother’s Delight‐Kw, Apomuden‐Ba, Kuffour‐Ba)
Disease and pest resistance	25.0	17.7	3 (Aragbe‐Ka, Dakata‐Ka, Dan Bakalori‐Ka)
Stores well	17.5	37.7	3 (Obare‐Ba, Dan China‐Ka, Alausa‐Kw)
Adapt to all soil types	0	10.0	1 (Alausa‐Kw)
Drought resistance	0	27.2	2 (Dan Izala‐Ka, Dan China‐Ka)
Health benefits	0	30.0	3 (Mother’s Delight‐Kw, Apomuden‐Ba, Kuffour‐Ba)
Less fertiliser usage	0	41.1	2 (Dakata‐Ka, Dan Barmawa‐Ka)
Vines available	0	100.0	1 (Obare‐Ba)

In individual interviews in Kwara, women preferred the white‐fleshed variety Pakurumon and the yellow‐fleshed variety Alausa and men preferred the yellow‐fleshed variety Alausa and the orange‐fleshed variety, Mother’s Delight. Similarly, the FGD data revealed that Alausa was the most grown variety for both genders. Men chose growing Alausa because of its marketability, short maturity period and low sugariness, whilst women desired its marketability. The men in Kwara FGDs reported that the orange‐fleshed sweetpotato variety, Mother’s Delight, had a high market value, but planting material was not readily available^3^.

In Bawku, the white‐fleshed local landrace Obare was the most preferred variety, irrespective of the producer’s gender. The orange‐fleshed varieties Kuffour (landrace) and Apomuden (bred) were also regarded highly by men and women, respectively. Similarly, FGDs revealed that Obare and Kuffour were the first and second most important sweetpotato varieties, for both sexes. Obare was preferred for its high yield, storability, yam‐like taste and generally high market demand (Table 1). Kuffour was preferred because of its high yield, early maturity and health benefits.

In summary, both men and women farmers in the study areas reported growing sweetpotato varieties that are high yielding (supported by pest and/or disease or drought resistance) and early maturing, meet their taste preferences regarding sugariness (which vary amongst consumer sub‐sets), are easily marketable and store well (Table 1). However, there are some optional attributes for growing sweetpotato varieties peculiar to either men or women. For instance, women farmers, exclusively, cited varieties with long vines for feeding animals, roots with short cooking times and better market prices. Only men cited growing varieties specifically for adaptation to all types of soil, tolerance to drought and lower fertiliser requirement, whose vines (planting materials) are readily available and roots have health benefits. The difference in attributes for growing sweetpotato that are peculiar to men and women could be attributed to either gender roles or the importance of sweetpotato to the livelihood and asset portfolio of men and women. For instance, growing varieties with long vines suggest the importance of livestock to women’s livelihoods, whilst preference for varieties with short cooking time reflects women’s role in caring for the home. The level of production of sweetpotato in the study area was higher for men (500‐3,899 Kg per season) than women (250‐835Kg per season). Thus, it is plausible that men seek to grow varieties with broad adaptation and lower fertiliser need, whose vines (planting materials) are readily available to support production at a higher scale to generate surplus for marketing.

### Fried sweetpotato process characterisation: steps and key processing parameters for ‘chunk fries’

**Figure 4:**
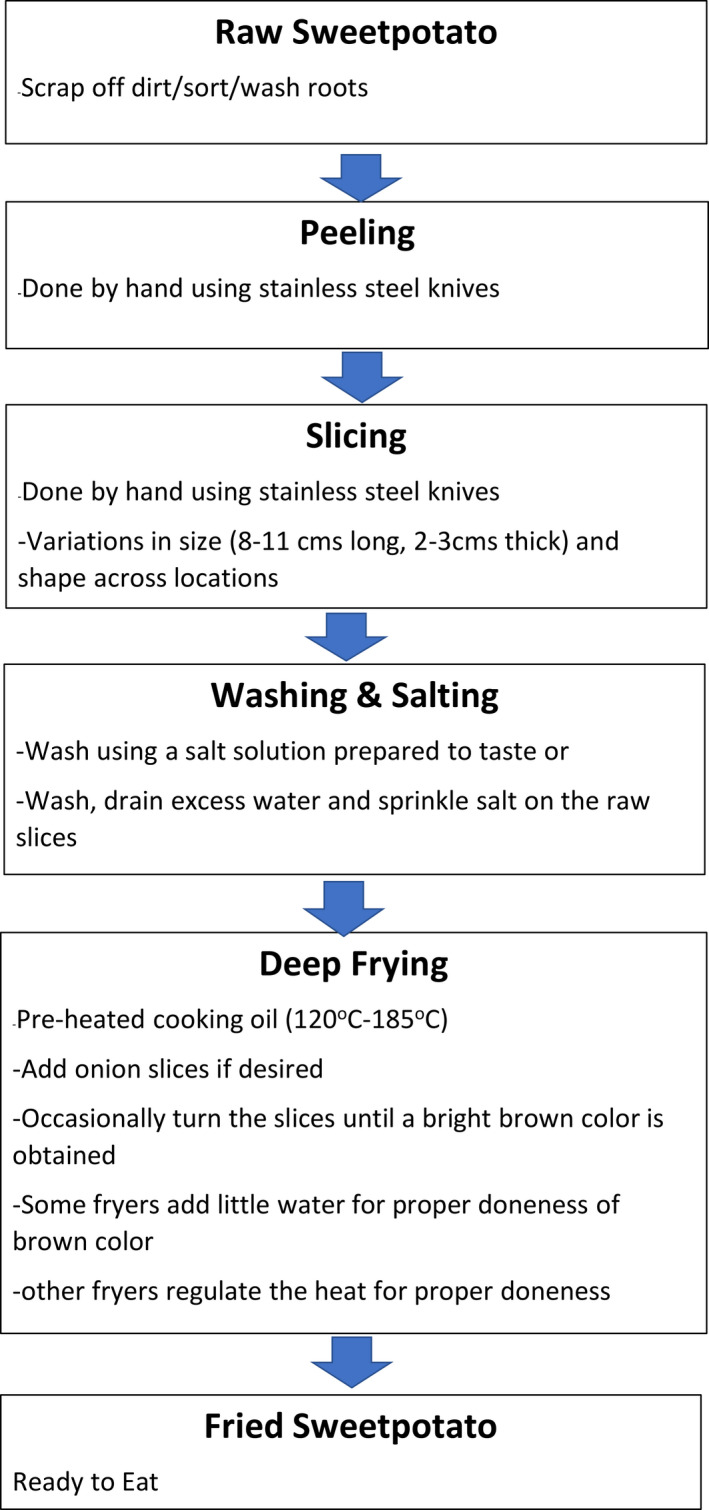
General flow chart for the fried sweetpotato processing components in Ghana and Nigeria.

Slight differences in either size or shape of the slices, salting technique and frying temperature were observed in the processing procedures (Figure 4) amongst the champion fryers across the eight locations. Fryers unanimously agreed that choice of variety determined the quality of fried sweetpotato product. Raw sweetpotato roots carefully chosen for frying have a regular shape, smooth skin, medium‐large root size (200–450 g) and no holes (indicative of weevil damage), making them easy to peel (Table 2A). Previous gender mapping of processed sweetpotato products in Africa has described peeling and slicing by hand using knives as time consuming activities often done by women (Mayanja & McEwan, 2015; Peters, 2015). Also, fryers sought raw sweetpotato roots with characteristics that indicate the likelihood of obtaining a high‐quality final fried product, like the firmness of the root when pressed. Firm roots indicate low moisture content which is associated with good frying characteristics such as requiring less frying time, absorbing less oil, crispness and increased shelf stability of fried products (Ziaiifar *et al*., 2008; Fetuga *et al*., 2013).

**Table 2:** Desirable characteristics at different processing units of the fried sweetpotato product

Characteristic	Why is it important
A. Characteristics of the raw sweetpotato root. Source: FGDs, Individual Interviews and Champion Fryers	
Firm	The roots are usually hard to slice, an indication of low moisture content which is associated with less frying time and subsequently using less oil
Smooth skin	Makes it easy to peel, that is in the absence of *lumps* (described as ‘bumpy nature’ by fryers) peeling takes less time and more slices are obtained
Regular shape	Makes it easy to peel and slice the root for frying, minimising excess weight loss during peeling
No holes	Makes the root easier to peel and yields more slices, that is less to discard; also an indication of no pest infestation and by inference, no rots
No off‐odours	An indication of no rots or pest infestation
Earliness	Ensures the crop is available at most of the times in the year; more than one harvest possible, especially in irrigated lowlands
Stores well	Reduces losses after the roots have been harvested, which ensures a longer marketing window and availability during off season
Medium‐big root size	Important to fryers because it is easier to peel and slice (with lesser risk of injuring oneself) and yields more slices
High yield	Ensures more slices from high yielding varieties per unit of land, thus more income
B. Characteristics of the peeled, sliced and washed sweetpotato. Source: Women FGDs and Champion Fryers	
Hard to slice	Indicates low moisture content and thus high dry matter content to give the desired end product characteristics
Does not stick to hand and not slippery	An important property during peeling and washing; further indication of low moisture content
Slices with uniform colour	Indicates that the final product will also have a uniform colour which is attractive to consumers
Slices with no or slight surface moisture	Gives an indication of low moisture content and thus, high dry matter, making it easy to fry
Slices not too sticky	Attribute indicated as important when peeling/slicing; an indication of low moisture content and thus, high dry matter
Slices with no off‐odours	Important attribute particularly during processing (slicing). Indicates no rottenness and to an extent, no pest infestation or disease
C. Characteristics of the fried sweetpotato final product. Source: FGDs, Champion Fryers and Consumers	
Uniform light brown (for white‐fleshed); uniform yellow with a brown tint (for yellow‐fleshed); uniform orange with a brown tint (for orange‐fleshed)	An indication fries are cooked – well fried and crisp; gives better consumer appeal
Dry/filling/satisfying	A desired consumer attribute – an indication of high dry matter and to an extent, mealiness (just like yam); sometimes expressed as ‘Spreads in the mouth’
Hard/strong, at first bite	A desired consumer attribute which is an indication of crispness
Crispy	A desired consumer attribute
Not soggy	An indication of crispness and being ‘dry’
Not oily	An indication of crispness and being ‘dry’. Such varieties do not consume too much oil
Moderately sugary	Sugariness – has to do with the flavour and more specifically, taste: a desired consumer attribute
Slightly sugary	A segment of consumers also preferred this as observed from preferences for Obare (slight sugariness) and Kuffour (moderate sugariness)

Fryers used several features observed during slicing to gauge raw sweetpotato roots for making the desired final fried sweetpotato product. For instance, the root being hard to slice or having no exudates (also referred to as ‘latex’) after peeling was associated with low moisture content (Table 2B). Preference for raw roots that are hard to slice suggests the need for low‐cost mechanisation for slicing to reduce drudgery in processing fried sweetpotato. Proper washing was another major unitof operation that champion fryers undertook to avoid having sand in the final product. There were two distinct salting practices in both countries: washing in a salt solution prepared to taste or sprinkling salt, by hand, on raw slices during drip drying.

Deep frying was the most important unit of operation determining the quality of fried sweetpotato product. Deep frying involves pre‐heating vegetable oil (temperatures varied from 120–185°C). Some fryers added slices of onions, usually to determine whether the oil is ready but also to improve the flavour of the final fried product. Slices of sweetpotato are added and occasionally turned until a bright, golden brown colour is achieved, and the oil stops bubbling, which takes around 5 minutes depending on oil temperature. Fryers had different methods of regulating oil temperature to ensure that the slices turned brown without burning, including adding cold water to the oil and removing fuel wood (to lower the temperature). The quality of the final fried product was defined by the fryer’s assessment of the appearance and the enjoyment they perceived their clients’ have expressed or would express. The attractiveness of sweetpotato final fries is partly determined by the flesh colour of the raw root. Three distinct appearance preferences were reported: either a uniform light brown, uniform yellow with a brown tint or uniform orange with a brown tint. Almost all consumers perceived enjoyment of the final fried product due to the taste (especially sugariness), crispness and satiation after eating (Table 2C).

### Characteristics of varieties with the best qualities for sweetpotato fried products

Attributes that characterised varieties considered most suitable for sweetpotato frying were consistent across sites, including:

- Medium to big root size,
- Little or no exudates (‘latex’) after peeling,
- Hard to slice roots,
- Short frying time,
- Attractiveness of the fried product,
- Exterior crispness of the fried chunks/slices,
- Not being either soggy or oily,
- Fried slices or chunks that are filling or satisfying when eaten.

In Bawku, Obare (dry matter 36%) was the variety of choice by the fryers, both before and during the diagnostic. It had a slightly sugary taste which was considered as the optimum sweetness, and the slices of the final product were crispy outside and soft inside. Other varieties evaluated by the fryers in Bawku had drawbacks. For example, the final fried product from Amuskwera (white‐fleshed, dry matter 27%) was considered hard immediately after frying but softens with time. Kuffour, which produced very attractive orange fried chunks, had a low ranking because it consumed a lot of oil due to its high moisture content (dry matter 25%).

In Kwara, the varieties Alausa (yellow‐fleshed, dry matter 40%) and Pakurumo (white‐fleshed, dry matter 32%) were the most suitable for frying mainly because of their big root size, moderate sugariness, hardness (low moisture content), good appearance, crispness of the final fried product and being satisfying (filling) when eaten. Other varieties in Kwara, like Aragbe (white‐fleshed, dry matter 30%), were not hard enough nor filling enough, whilst Elege (white‐fleshed, dry matter 36%) was condemned for easily shrivelling if not processed immediately after harvest. Despite the attractive golden colour of orange‐fleshed Mother’s Delight (dry matter 23%) after frying, the final product was not crispy, needing too much oil to fry.

In Kano, Dan Izala (white‐fleshed, dry matter 35%), the most popular variety for the fryers produced low exudates (latex) after peeling, had a short frying time, did not soak oil and produced mealy and crispy chunks. Surprisingly, the two least suitable varieties in Kano, Dan Silver (white‐fleshed) and Mother’s Delight (orange‐fleshed), had contrasting characteristics. Dan Silver (dry matter 33%) produced almost no exudates after peeling and had a short frying time to produce hard and very crispy fries; whilst Mother’s Delight had a lot of exudates after peeling, a long frying time and the fries were too soft and soggy. This highlights that multiple traits in specific combinations of attributes will influence overall suitability for frying. Table 3 captures fryer perspectives in Bawku on the different varieties. Tables summarising champion fryer perspectives for each variety in Kano and Kwara are available upon request.

**Table 3:** Champion fryer perspectives prior to and during diagnostic in Bawku, Upper East Region Ghana

Variety	Weighted score for preference*	Champion fryer perspectives	
		Prior to diagnostic	During diagnostic
Obare	32	Most suitable for frying	Texture just right in terms of hardness/ dryness, sweetness just right, crispy outside and soft inside.
Purupuru	21	Very soft, soaks oil during frying; consumers complain it is not sweet and inside is not compact (not mealy)	Fries well and the colour change is good; however, it is too soft (moisture content high); outside not dry enough; the feel could be better if stored for a few days before processing.
Kuffour	16	Too sweet, low demand with adult markets [mostly preferred by school children]; high moisture content	Irregular shape and pest damage make peeling and slicing difficult. Very sugary taste; too much moisture; consumes oil and soft. However, the final colour of cooked product is good.
Amuskwera	11	Does not dry, no taste, high moisture content, has no aroma, has no flavour, low demand when fried	Good appearance when fried (brightness of colour). Hard as desired immediately after frying but softens with time.

* Weighted preference scores obtained by multiplying the frequency of the fryers who ranked a variety as most preferred (1st position) by 4, and the least preferred variety by 1.

Most of the desirable quality attributes highlighted by the stakeholders such as high dry matter content, crispness, mealiness and the short frying time without soaking up a lot of oil are likely to be functions of starch properties. For instance, a strong positive correlation was reported between amylose content and crispness in several crop starches, whilst it was negatively correlated with oil content of the fried products (Zhang *et al*., 2014). Crispness is related to the mechanical properties of the crust structure which is formed when amylose leaches from the swollen starch granules during frying. The resulting amylose gel pasting becomes crispy when dehydrated (Lisinska & Golubowska, 2005). Crispness is lost due to changes in the mechanical properties of the solid matrix, which is caused by a complex process in which temperature, water and oil play a role (Ziaiifar *et al*., 2008). This implies that breeding efforts should prioritise relating physico‐chemical properties of raw and fried sweetpotato to sensory properties and consumer acceptance. This will assist breeding programmes to select varieties that make better quality sweetpotato fried products. Acceptable levels of sugary taste, which were noted to vary amongst consumers, probably relate to amylases, which vary amongst genotypes and are activated during cooking (Kitahara *et al*., 2017). Amylase activity during cooking results in starch hydrolysis, maltose accumulation and associated production of thermally induced flavour compounds particularly related to the Maillard reaction (Kays & Wang, 2000). A further element that may contribute to the product profile is preference for low exudation of latex during peeling and slicing. Informants in Kano commented on the superiority of cultivar Dan Izala, which was low in sticky exudates, amongst other attributes. Latex production in sweetpotato is known to vary with genotype, with maturity, and postharvest interval (Brabet et al., 1999; Nedunchezhiyan and Ray, 2010).

## Conclusions

Up to 75% of the sweetpotato produced in selected communities of Kano and Kwara States in Nigeria, and Bawku in the Upper East Region of Ghana was consumed as chunk fries in urban centres. Given the rapid urbanisation occurring in West Africa, fast foods are becoming more and more popular, necessitating sweetpotato varieties that are suitable for frying. However, none of the current varieties is grown only for processing fried product, as sweetpotato is also grown for home consumption as a boiled root. Communities largely appreciate the nutritious benefits of orange‐fleshed sweetpotato, but those varieties encountered in this study were not considered suitable for chunk fries.

Champion fryer diagnosis enabled us to understand the characteristics of varieties suitable for frying. Suitable raw roots should be hard when pressed with the finger, have a regular shape and a smooth skin without any grooves, and no off‐odours or holes. Most of these characteristics are geared towards easy handling, minimising loss from pests and rots, but also ensuring a quality final product. During peeling and slicing, raw roots should be hard to slice, whilst after slicing, the surface should be almost dry and not sticky. Hard to slice roots connote both high dry matter and mealiness. Stickiness and a moist surface indicate high moisture content, associated with excessive oil absorption during frying. Preferences on the uniformity of the colour of the final fried product vary. Some fryers said that clients prefer a product with a uniform colour with a brown tint and not be soggy or oily. Others prefer the attractive orange chunky fries. Additionally, the product should be crispy, with a slightly sugary taste and mealy internal texture.

Crispness, mealiness and short frying time of the chunks with limited oil absorption are functions of the starch properties of the sweetpotato. Breeding programmes have to consider these attributes so as to develop varieties suitable for frying and acceptable to consumers. Exposure to high temperatures during frying causes a limited loss of ß‐carotene, which is well compensated for by the increased bio‐accessibility of β‐carotene in fried slices (Kourouma *et al*., 2019). This implies that developing orange‐fleshed varieties suitable for frying could improve uptake and nutritional benefits of the crop. Defining the characteristics that make a variety suitable for frying can be useful for refining the trait‐introgression pipeline for fried sweetpotato, which involves assembling germplasm, developing high‐throughput phenotyping tools and incorporating traits into selection. Assembling germplasm with attributes that make a variety fit for frying will provide a good foundation for developing such new varieties. This study identifies traits that could broadly guide the germplasm assembly like high dry matter(>30%) and high starch content of raw roots (>60% on dry weight basis), which are already routinely measured by sweetpotato breeding programmes, as are raw sugar types (fructose, sucrose and glucose) and sugars in boiled roots (including maltose) in West Africa only. Developing high‐throughput phenotyping tools will require identifying how attributes for frying like crispness, mealiness and the short frying time without soaking up a lot of oil are associated with physico‐chemical parameters of starch, which are influenced by varying amylose to amylopectin ratios (Oladebeye *et al*., 2009). This suggests that breeding programmes could routinely predict the amylose content using near‐infrared spectroscopy (NIRS) models.

Breeding programmes will also need to re‐arrange components of the pipeline to engage fryers in variety development. The most suitable varieties for frying identified during the fryer diagnostic component, like Obare in Bawku, Dan Izala in Kano State and Alausa in Kwara State can be used as check clones to benchmark suitability of clones for frying along the breeding pipeline. Further work is needed to understand the relevance of our findings about chunk fries to other forms such as French fries and chips, where more advanced understanding of selection criteria for quality may help to guide breeding for chunk fry quality (Carey *et al*, 2021).

## Author Contribution

**Reuben Ssali:** Data curation (lead); Formal analysis (lead); Investigation (equal); Supervision (equal); Writing‐original draft (lead); Writing‐review & editing (supporting). **Edward E. Carey:** Conceptualization (supporting); Investigation (equal); Methodology (supporting); Supervision (equal); Writing‐original draft (supporting); Writing‐review & editing (equal). **Simon Imoro:** Data curation (lead); Investigation (supporting); Software (supporting). **Jan W. Low:** Project administration (lead); Resources (lead); Supervision (supporting); Writing‐review & editing (equal). **Eric Kuuna Dery:** Investigation (equal); Validation (supporting); Writing‐original draft (supporting). **Abena Boakye:** Investigation (equal); Supervision (supporting); Validation (supporting); Writing‐original draft (supporting). **IBOK NSA ODURO:** Supervision (supporting); Writing‐review & editing (supporting). **Rachel M. Omodamiro:** Investigation (equal); Supervision (equal); Writing‐original draft (supporting). **Hauwa Ladi Yusuf:** Investigation (equal); Writing‐original draft (supporting). **Eunice Etwire:** Investigation (equal); Writing‐original draft (supporting). **Abigail O. Iyilade:** Investigation (equal); Writing‐original draft (supporting). **Souleimane Adekambe:** Investigation (equal); Writing‐original draft (supporting). **Abdullahi Ali:** Investigation (equal); Writing‐original draft (supporting). **Muhammad Haliru:** Investigation (equal); Writing‐original draft (supporting). **Prince Maxwell Etwire:** Investigation (equal); Writing‐original draft (supporting).

## Conflict of interest

The authors declare no conflict of interest in this work.

## Ethical guidelines

Research teams operated under the aegis of national research programme guidelines. Participants were informed about the study, they could stop the interview at any point, and written consent from sensory panellists and from consumers participating in this study were obtained and the research respected the rules of voluntary participation and anonymity. Food samples were prepared according to good hygiene and local practices.

### Peer review

The peer review history for this article is available at https://publons.com/publon/10.1111/ijfs.14764.

## Data Availability

Data available on request from the authors.

## Notes

^1^ Key informants were opinion leaders in the community, who happened to be only men in the communities visited.

^2^ A very . It is a field dominated by women, even in predominantly Muslim Kano and Bawku.

^3^ Mother’s Delight was the first released OFSP variety in Nigeria and the planting material faces challenges in terms of vine survival after the dry season. Farmers have to return to vine multipliers to replenish their planting material.
